# Diabetic Striatopathy: A Case Report of Two Distinct Presentations in Elderly Patients

**DOI:** 10.7759/cureus.72284

**Published:** 2024-10-24

**Authors:** Paula A Calvo, Raquel Flores

**Affiliations:** 1 Endocrinology, Diabetes and Metabolism, Unidade Local de Saúde Lisboa Ocidental - Hospital de Egas Moniz, Lisbon, PRT; 2 Internal Medicine, Unidade Local de Saúde Lisboa Ocidental - Hospital de Egas Moniz, Lisbon, PRT

**Keywords:** ballism, chorea, diabetic, hyperglycemia, striatopathy

## Abstract

Movement disorders associated with diabetes mellitus (DM) are rare. The diagnosis of diabetic striatopathy (DS) is based on the presence of a triad characterized by hyperglycemia, hemiballismus/chorea, and hypersignal of the basal ganglia on T1-weighted MRI. In most cases, treatment involves glycemic control. DS should be considered a differential diagnosis in episodes of extrapyramidal movements, especially when associated with hyperglycemia in the elderly. In this paper, we present two cases of DS.

## Introduction

Diabetic striatopathy (DS) is a rare complication of diabetes mellitus (DM), affecting 1 in 100,000 people [1]. It was first described by Bedwell, primarily in elderly women [2]. Although rare, DS is the second most common cause of chorea and hemiballismus, following cerebrovascular events involving the basal ganglia [1].

The term "diabetic striatopathy" was introduced in the last decade to describe a hyperglycemic state associated with involuntary limb movements, usually chorea or ballism, and a unique, reversible abnormality of the basal ganglia on computed tomography (CT) and magnetic resonance imaging (MRI). In recent years, the use of this term has expanded to include cases with hyperglycemia and either involuntary movements or abnormal MRI findings [3].

This condition is most commonly reported in patients with type 2 diabetes (DM2) and poor metabolic control, particularly older Asian women [1,3]. However, cases have also been reported in Caucasian and Hispanic populations [4,5]. Treatment primarily involves glycemic control, although neuroleptic agents may be used in some cases [6].

The anatomical location and pathophysiological mechanism of DS are not yet fully understood [7]. It has been suggested that DS may have a microvascular etiology.

We present two cases of DS: one in a man without a known history of DM and another in a woman with a long-standing history of diabetes.

## Case presentation

Clinical case 1

**Figure 1:**
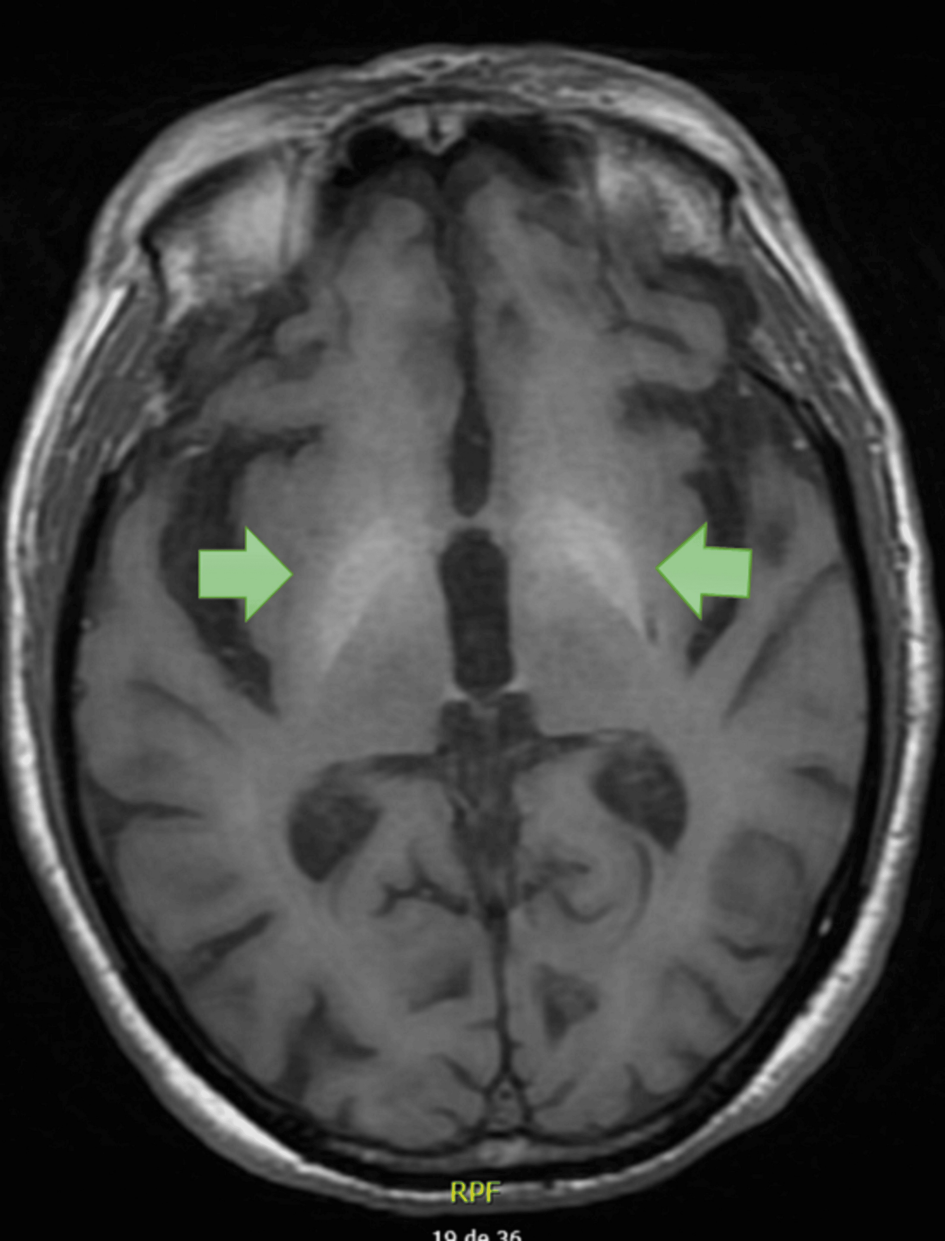
MRI of Patient 1 showing T1 hypersignal in the lenticular nuclei.

A 79-year-old male with a history of arterial hypertension, dyslipidemia, and benign prostatic hyperplasia was admitted to the emergency room with complaints of five days of polydipsia, polyuria, speech disturbance, and involuntary movements of the left limbs. The movements were dystonic and choreoathetoid, more severe in the upper limb. Bloodwork revealed hyperglycemia of 890 mg/dL (49.4 mmol/L) with negative ketonemia and an HbA1c of 14.3% (Table 1). An angio-CT scan ruled out ischemic or hemorrhagic stroke. An MRI showed a mild hypersignal in both lenticular nuclei, without involvement of the putaminal or caudate nuclei (Figure 1). He was started on intensive insulin therapy, resulting in the complete remission of neurological symptoms once glycemic control was achieved. During follow-up, he discontinued insulin and began treatment with oral hypoglycemic agents. To date, he has not experienced new episodes of involuntary movements.

Clinical case 2

An 84-year-old woman with a long-standing history of DM2, with deficient metabolic control and complicated by coronary artery disease and cerebrovascular disease, was admitted to the emergency room with a one-week history of hyperglycemia, urinary incontinence, psychomotor agitation, and eventual unconsciousness. On physical examination at admission, she exhibited conjugated eye deviation to the left side and choreatic movements of the left limbs.

**Figure 2:**
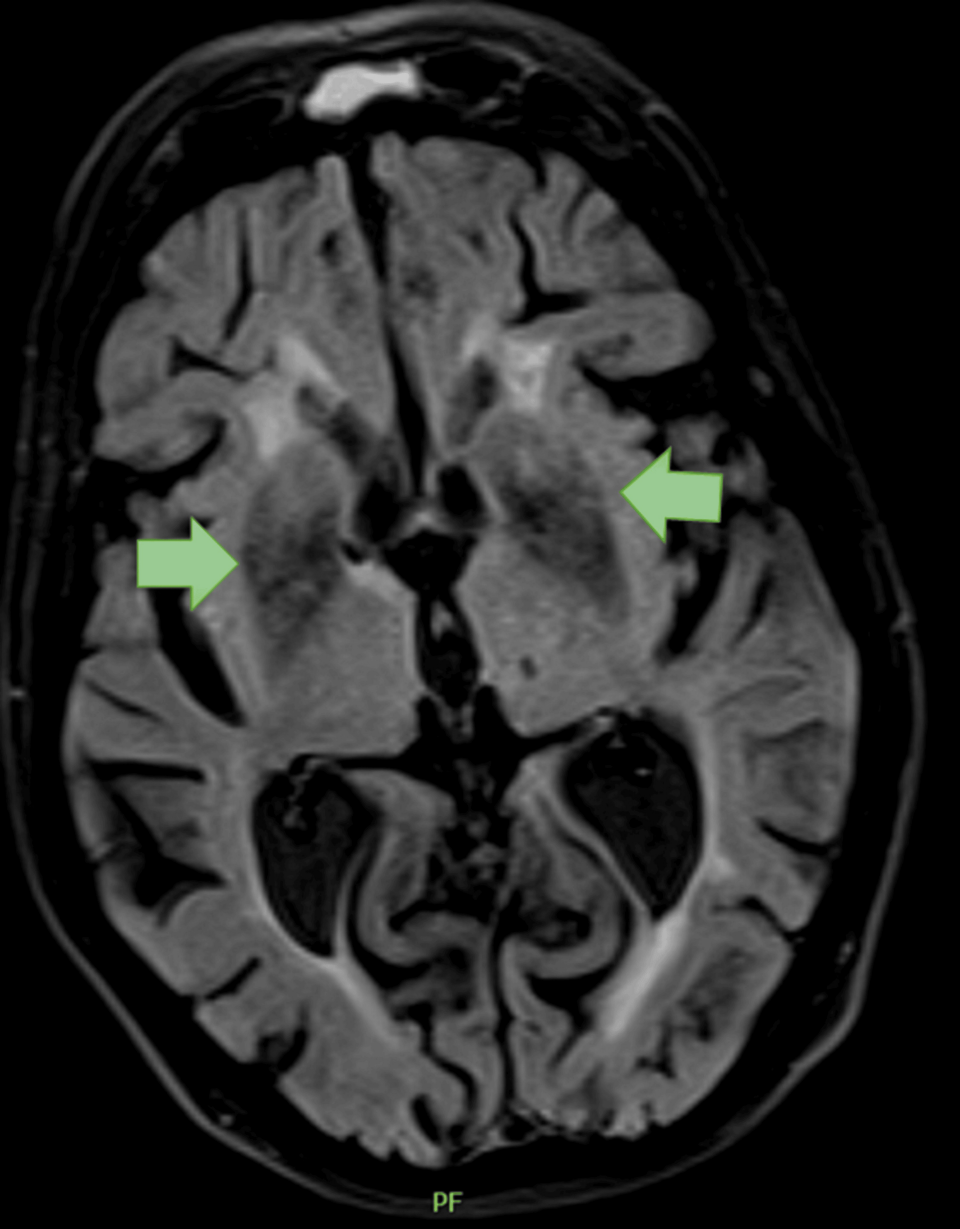
MRI of Patient 2 showing hyposignality in the corpus striatum in FLAIR T2. FLAIR: fluid-attenuated inversion recovery.

Bloodwork revealed hyperglycemia of 799 mg/dL, an HbA1c of 11% (Table 1), elevated inflammatory markers, and leukocyturia. An MRI showed hyposignal involving the corpus striatum bilaterally, extending to the medial portion of the cerebral crus on T2 and T2-fluid-attenuated inversion recovery (FLAIR) sequences, along with a mild hypersignal in the basal nuclei on T1 (Figure 2).

**Table 1:** Laboratory findings related to the metabolic state in both cases.

Laboratory parameter	Case 1	Case 2	Normal range
Glycemia (mg/dL)	890	799	74-106 mg/dL
HbA1c	14.30%	11%	<5.7%

The patient was initially treated with diazepam and insulin, resulting in partial clinical improvement, though extrapyramidal symptoms persisted despite metabolic correction. Consequently, tetrabenazine was initiated, leading to the full remission of involuntary movements. Treatment with tetrabenazine was discontinued after two months, with clinical stability maintained.

A year later, the patient was readmitted with an episode of diabetic hyperosmolar syndrome. During this episode, she also presented with choreoathetoid movements in both upper limbs (Video 1). Tetrabenazine was reintroduced, but the patient continued to experience mild myoclonus in the left limbs after discharge. During follow-up, the patient was asymptomatic after three months of tetrabenazine treatment, and the medication was discontinued without any recurrence of choreatic movements following withdrawal.

**Video 1:** Involuntary movements of Patient 2. The patient before starting on tetrabenazine showed choreoic movements of her left and right upper limbs.

## Discussion

DS is more common in patients with long-standing diabetes, usually with poor metabolic control. The underlying vascular disease, and therefore vascular insufficiency, makes the diabetic brain more susceptible to DS due to the rich basal ganglia irrigation and high metabolic demands [7]. Ischemia-induced dysfunction of gamma-aminobutyric acid (GABA) projection neurons is a proposed mechanism [8]. However, there also seems to be reduced inhibition in the thalamus and the corpus striatum caused by diminished GABA levels due to high encephalic acetate and glucose levels. This results from a disruption of the blood-brain barrier caused by plasma hyperviscosity [9], leading to disinhibition of the basal ganglia and causing involuntary movements [3]. This mechanism may explain the occurrence of DS in diabetic patients with mild diabetes and no previously known vascular complications.

In both of our cases, the MRI showed a hypersignal on T1 in the basal nuclei. In a study of 153 cases, the hypersignal was often found in the putamen and/or caudate nucleus and less frequently in the globus pallidus [3]. To differentiate this hypersignal from hypertensive hemorrhage, it is important to note the absence of mass effect and the sparing of the internal capsule [10]. The hypersignal appears soon after the onset of DS and may become more pronounced even after symptom remission. Although the relationship between imaging findings and the clinical course is not fully understood, it is believed to reflect neuronal loss, dysfunction, gliosis, and ischemia in the basal ganglia [11]. Neither of our cases underwent a follow-up MRI due to clinical stability.

A case report described a parenchymal transcranial sonography (PTcS) that revealed hyperechogenicity in the right lenticular nucleus, with a subsequent MRI showing hypersignal in the ipsilateral striatum on T1 [12]. Although PTcS was not performed in our cases, it could be a useful diagnostic tool where immediate MRI is unavailable.

While glycemic control and hydration are the cornerstones of DS treatment, up to 75% of cases are resistant to glycemic control alone and require modulation of GABA-ergic and dopaminergic transmission [3,13]. Antipsychotics like tetrabenazine, a vesicular monoamine transporter 2 inhibitor and mild dopamine receptor blocker that increases dopamine degradation by monoamine oxidases, are indicated in such cases [10,13]. In most cases, antipsychotics can be discontinued after a few months with full remission [3,5,10]. However, the factors influencing the resolution time of clinical dyskinesias remain inconsistent in the literature [5].

## Conclusions

DS is a rare complication of DM; however, it should be considered a differential diagnosis in episodes of extrapyramidal movements, especially when associated with hyperglycemia in the elderly, given that it is the second most common cause of extrapyramidal disorders after vascular disease. In most cases, the movements cease with proper metabolic control.
